# Comparative Efficacy and Acceptability of Psychotherapies for Self-harm and Suicidal Behavior Among Children and Adolescents: A Systematic Review and Network Meta-analysis

**DOI:** 10.1001/jamanetworkopen.2021.6614

**Published:** 2021-04-16

**Authors:** Anees Bahji, Matthew Pierce, Jennifer Wong, Johanne N. Roberge, Iliana Ortega, Scott Patten

**Affiliations:** 1 Department of Psychiatry, University of Calgary, Calgary, Alberta, Canada; 2 Department of Psychiatry, Queen’s University, Kingston, Ontario, Canada; 3 Division of Child and Youth Mental Health, Queen’s University, Kingston, Ontario, Canada; 4 Department of Community Health Sciences, University of Calgary, Calgary, Alberta, Canada

## Abstract

**Importance:**

Self-harm and suicidal behavior are associated with substantial morbidity and mortality among children and adolescents. The comparative performance of psychotherapies for suicidality is unclear because few head-to-head clinical trials have been conducted.

**Objective:**

To compare the efficacy of psychotherapies for the treatment of self-harm and suicidality among children and adolescents.

**Data Sources:**

Four major bibliographic databases (PubMed, MEDLINE, PsycINFO, and Embase) were searched for clinical trials comparing psychotherapy with control conditions from inception to September 2020.

**Study Selection:**

Randomized clinical trials comparing psychotherapies for suicidality and/or self-harm with control conditions among children and adolescents were included after a blinded review by 3 independent reviewers (A.B., M.P., and J.W.).

**Data Extraction and Synthesis:**

The Preferred Reporting Items for Systematic Reviews and Meta-analyses (PRISMA) reporting guideline was followed for data abstraction, and the Cochrane risk of bias tool was used to evaluate study-level risk of bias. Data abstraction was performed by 1 reviewer (A.B.) and confirmed by 2 independent blinded reviewers (J.W. and M.P.). Data were analyzed from October 15, 2020, to February 15, 2021.

**Main Outcomes and Measures:**

The primary outcomes were dichotomized self-harm and retention in treatment. The secondary outcomes were dichotomized all-cause treatment discontinuation and scores on instruments measuring suicidal ideation and depressive symptoms. Effect sizes were pooled using frequentist random-effects network meta-analysis models to generate summary odds ratios (ORs) and Cohen *d* standardized mean differences (SMDs). Negative Cohen *d* SMDs or ORs less than 1 indicated that the treatment reduced the parameter of interest relative to the control condition (eg, signifying a beneficial association with suicidal ideation).

**Results:**

The systematic search generated 1272 unique records. Of those, 44 randomized clinical trials (5406 total participants; 4109 female participants [76.0%]) from 49 articles were selected (5 follow-up studies were merged with their primary clinical trials to avoid publication bias). The selected clinical trials spanned January 1, 1995, to December 31, 2020. The median duration of treatment was 3 months (range, 0.25-12.00 months), and the median follow-up period was 12 months (range, 1-36 months). None of the investigated psychotherapies were associated with increases in study withdrawals or improvements in retention in treatment compared with treatment as usual. Dialectical behavioral therapies were associated with reductions in self-harm (OR, 0.28; 95% CI, 0.12-0.64) and suicidal ideation (Cohen *d* SMD, −0.71; 95% CI, −1.19 to −0.23) at the end of treatment, while mentalization-based therapies were associated with decreases in self-harm (OR, 0.38; 95% CI, 0.15-0.97) and suicidal ideation (Cohen *d* SMD, −1.22; 95% CI, −2.18 to −0.26) at the end of follow-up. The quality of evidence was downgraded because of high risk of bias overall, heterogeneity, publication bias, inconsistency, and imprecision.

**Conclusions and Relevance:**

Although some psychotherapeutic modalities appear to be acceptable and efficacious for reducing self-harm and suicidality among children and adolescents, methodological issues and high risk of bias prevent a consistent estimate of their comparative performance.

## Key Points

**Question:**

What are the comparative efficacies and acceptability of psychosocial interventions for the treatment of self-harm and suicidality among children and adolescents?

**Findings:**

In this systematic review and network meta-analysis of pooled data from 44 randomized clinical trials of psychotherapies for children and adolescents that involved 5406 total participants, the investigated psychotherapies were found to be acceptable to patients, but the evidence was inconsistent with regard to self-harm and suicidality measures across therapeutic modalities.

**Meaning:**

The findings indicate that, although some psychotherapeutic modalities appeared to be acceptable and efficacious for reducing self-harm and suicidality among children and adolescents, methodological issues and high risk of bias suggest a need for additional randomized clinical trials.

## Teaser

This systematic review and network meta-analysis uses pooled data from randomized clinical trials to compare the efficacy and acceptability of psychotherapies for the treatment of self-harm and suicidal behavior among children and adolescents.

## Introduction

Over the past 2 decades, there has been an increase in research exploring diverse aspects of self-harm and suicidal behavior among youths.^1^ Self-harm appears to peak in adolescence,^2^ with recent global surveys indicating that between 10% and 20% of adolescents reported past-year suicidal ideation and suicide attempts.^3^ In addition to sex and gender considerations,^4^ genetic vulnerability and several psychiatric, psychosocial, familial, and cultural factors may mediate suicide risk.^5^ Substance use, particularly cannabis, has also been implicated as a risk factor for self-harm and mortality risk among young adults.^6,7^

Despite the advances in research on the prevalence, correlates, classification, and function of self-harm and suicidal behaviors, there has been limited progress in reducing suicide rates for almost 60 years.^8,9^ Self-harm and suicidality among youths continue to be substantial burdens for patients, families, communities, and health systems.^1,10,11,12,13,14^ Evidence-based self-harm and suicide prevention efforts aimed at young people are needed.^5,15^

At present, there are insufficient data from randomized clinical trials (RCTs) to recommend targeted pharmacological treatments for self-harm or suicidal behavior in youths. However, some nonpharmacological interventions, including psychotherapies, appear to improve some aspects of suicidal behavior. Several meta-analyses have synthesized data from RCTs examining psychotherapies for self-harm and suicidality in youth populations. Ougrin et al^8^ found the largest effect sizes with dialectical behavioral therapy (DBT), cognitive behavioral therapy (CBT), and mentalization-based therapy (MBT). Nonetheless, they noted a lack of independent replications of efficacy for any intervention.

Hawton et al^15^ reported preliminary data indicating that MBT may be associated with reductions in self-harm and recommended further evaluation of therapeutic assessment and DBT. However, no evidence was found to indicate that group-based therapies, compliance enhancement, CBT, family-based therapy, or provision of an emergency card was associated with decreases in suicidal behaviors. Robinson et al^16^ reported no differences between treatment and control groups across 15 RCTs, with the exception of 1 study that compared CBT with treatment as usual. Storebø et al^17^ found that DBT and MBT had some beneficial consequences for reducing self-harm among individuals with borderline personality disorder (BPD) but noted that these conclusions were based on low-quality evidence. Jørgensen et al^18^ reported a significant association between DBT and self-harm at the end of treatment compared with control interventions but no association between cognitive analytic therapy or MBT and reductions in self-harm among adolescents with BPD or BPD features compared with treatment as usual, emphasizing the need for more high-quality clinical trials with larger samples.

Kothgassner et al^2^ found that the pooling of psychological treatments was associated with improvements in self-harm, suicidal ideation, and depressive symptoms compared with active control conditions, with subgroup analyses indicating that DBT and family-based therapy may be associated with decreases in self-harm and suicidal ideation. Previous authors of systematic reviews have cited the small number of RCTs, limited direct comparisons between treatments, low quality of evidence, and lack of independent replication of individual RCT findings as key limitations.^2,8^ Given the inconsistency across previous reviews, the most appropriate type of psychotherapy for the treatment of adolescents who present with self-harm or suicidality remains unclear.

An alternative approach, termed network meta-analysis (NMA), might alleviate some of these previous challenges, particularly the shortage of head-to-head studies.^19,20,21^ An NMA is a meta-analysis of multiple treatments that simultaneously compares treatments across direct and indirect evidence sources in a single network.^22^ Network meta-analysis can be used to pool the samples across many small RCTs to increase the power for detecting differences across outcomes. Network meta-analyses may be preferable to standard meta-analyses in some situations, as the network's indirect comparisons can mitigate study-specific biases that are not identifiable in head-to-head RCTs.^22^ A network meta-analysis can also incorporate more data into the analysis, allowing researchers to tackle the bigger picture, while a traditional meta-analysis often provides a fragmented view.^22^ However, the valid application of NMA depends on the satisfaction of several statistical requirements, such as a similar distribution of effect modifiers across clinical trials and comparisons.^20^ The present NMA aimed to reexamine the comparative efficacy and safety of psychotherapies for the treatment of self-harm and suicidal behaviors among children and adolescents.

## Methods

This review was registered with the Open Science Framework (https://osf.io/zcwvk) and adhered to the Preferred Reporting Items for Systematic Reviews and Meta-analyses (PRISMA) reporting guideline and its extension for NMAs.^23,24^

### Eligibility Criteria

We used the populations-interventions-comparators-outcomes-study design framework to define review eligibility. We considered RCTs that measured self-harm or suicidal behavior among children or adolescents aged 10 to 19 years. We defined self-harm as any intentional injury to oneself, regardless of suicidal motivation.^2^ To categorize interventions, we coded therapy protocols using the following groups: brief intervention, cognitive analytic therapy, CBT, DBT, family-based therapy, interpersonal therapy, MBT, mode deactivation therapy, supportive therapy, and short-term psychoanalytic psychotherapy (eTable 1 in the Supplement). Clinical trials blending 3 or more modalities were categorized as eclectic therapies.

To facilitate our analyses, we collapsed some interventions into larger categories. For example, brief motivational interviewing sessions, hospital admission tokens, brief app-based interventions, and youth-nominated support teams were categorized as brief interventions. Subcomponents of established psychotherapy were collapsed into the parent modality (eg, emotion regulation training and mindfulness interventions into DBT), and variants of an established modality were collapsed into the main classification (eg, MBT for adolescents into MBT). We defined nondirective nonspecific interventions as supportive therapy. Therapies were categorized as either individual or group rather than considering group therapy as a separate modality. We considered treatment as usual, enhanced usual care, waitlist control, and active comparators; however, we collapsed enhanced usual care into treatment as usual.

The primary outcomes were self-harm frequency (participants with ≥1 deliberate episodes of self-harm, including suicide attempts and nonsuicidal self-injury) and retention in treatment (participants who completed the primary treatment protocol). Secondary outcomes were study withdrawals (the number of participants who withdrew from the clinical trial for any reason) and suicidal ideation and depression severity, measured using clinician- or self-rated instruments. We excluded nonrandomized designs, crossover RCTs, and studies with missing or unobtainable data.

### Search Strategy, Selection, and Data Collection

We developed a comprehensive search strategy in PubMed, MEDLINE, Embase, and PsycINFO from the date of their inception to September 15, 2021 (eTable 2 in the Supplement). Search terms included *self-harm*, *self-injury*, *suicidal ideation*, or *suicidal behavior* and *therapy* or *intervention*. We reviewed the bibliographies of included records and previous reviews to supplement the electronic search.

Our review relied on Covidence, a web-based systematic review manager,^25,26^ to facilitate study selection by 2 investigators (A.B. and M.P.) who independently screened all records for the eligibility criteria by title and/or abstract and full text. Discrepancies were resolved through consensus.

Three reviewers (A.B., M.P., and J.W.) independently abstracted data and performed quality assessments using a spreadsheet (Microsoft Excel; Microsoft Corp). Extracted variables included sample size, demographic characteristics, intervention characteristics (modality and number of sessions), outcome measures, study name and authors, study location, and treatment duration and follow-up.

### Risk of Bias

To evaluate risk of bias within studies, 3 reviewers (A.B., M.P., and J.W.) independently appraised RCT quality using the Cochrane risk of bias tool,^27^ which assigns a low, high, or unclear rating to 6 domains: randomization, allocation concealment, blinding of participants, blinding of evaluators, incomplete outcome reporting, and selective reporting. We also considered allegiance, adherence, and attention biases.^18^ Allegiance bias occurs when the developer of a treatment is also an RCT investigator. Adherence bias concerns the fidelity of a treatment to protocol. Attention bias is produced by discrepant therapy doses (ie, sessions) between RCT arms. Overall study-level bias was considered high if any individual domain received a high score or had 2 or more unclear fields.

To assess the risk of bias across studies, we evaluated publication bias by graphing funnel plots^28^ and applying the Egger test.^29^ We used Grading of Recommendations Assessment, Development and Evaluation (GRADE) guidelines,^30^ and we downgraded the quality of evidence if we detected a high risk of bias, imprecision in outcomes, or heterogeneity.

### Summary Measures and Statistical Analysis

We used Cohen *d* standardized mean differences (SMDs) and odds ratios (ORs) to summarize effect sizes for continuous and dichotomous variables. Standardized mean differences of 0.2, 0.5, and 0.8 corresponded to small, medium, and large effect sizes.^31^ Negative Cohen *d* SMDs or ORs less than 1 indicated that the treatment reduced the parameter of interest relative to the control condition (eg, signifying a beneficial effect for suicidal ideation).^31,32^

We followed the same analytic approaches used in previous NMAs of studies examining psychiatric disorders (eMethods in the Supplement).^33,34,35,36,37,38^ We used the RStudio netmeta package, version 3.5.1 (RStudio).^39,40^ Forest plots were graphed for each outcome measure (self-harm, retention in treatment, study withdrawals, suicidality, and depression), and treatment rankings were created to represent each therapy’s effect size compared with treatment as usual. To preserve randomization, we used frequentist random-effects models, which accommodate different measures for the same outcome (eg, alternative instruments measuring suicidal ideation).^41^ To maximize available data, outcomes presented as dichotomous were pooled with continuous data using an inverse variance method. We assumed a jointly randomizable network, in which participants were equally likely to be randomized to any of the treatments.^21,41,42,43^ To determine NMA goodness of fit, transitivity (the extent of network heterogeneity) and consistency (the extent of agreement between direct and indirect comparisons)^44^ were assessed. To quantify transitivity, τ^2^ (total variation) and *I*^2^ (percentage of τ^2^ not caused by random error) were measured, with higher values indicating more heterogeneity.^45,46^ The Cochrane *Q* statistic was used to evaluate consistency, with the assumption of a full design-by-treatment interaction random-effects model; *P* > .05 indicated that the model was consistent. Dual analyses were conducted by distinguishing outcomes at the end of treatment from outcomes at the end of follow-up.

Network-level subgroup or meta-regression analyses could not be performed owing to limitations in the currently available RStudio packages. Data were analyzed from October 15, 2020, to February 15, 2021.

## Results

### Study Selection and Characteristics

**Figure 1.**
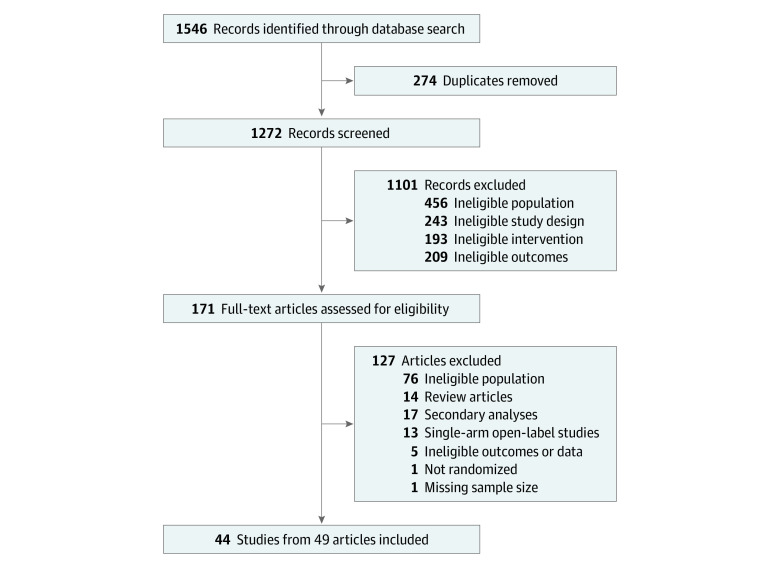
Preferred Reporting Items for Systematic Reviews and Meta-analyses (PRISMA) Flowchart of Study Selection Process

The systematic search identified 1272 unique records (Figure 1). After exclusion of 1101 records for ineligible study population, design, intervention, and/or outcomes, 171 full-text articles were assessed for eligibility. Of those, 44 RCTs (5406 total participants; 4109 female participants [76.0%]) from 49 articles were selected. To avoid publication bias, we merged 5 follow-up RCTs^47,48,49,50,51^ with their primary clinical trials.^52,53,54,55^

**Figure 2.**
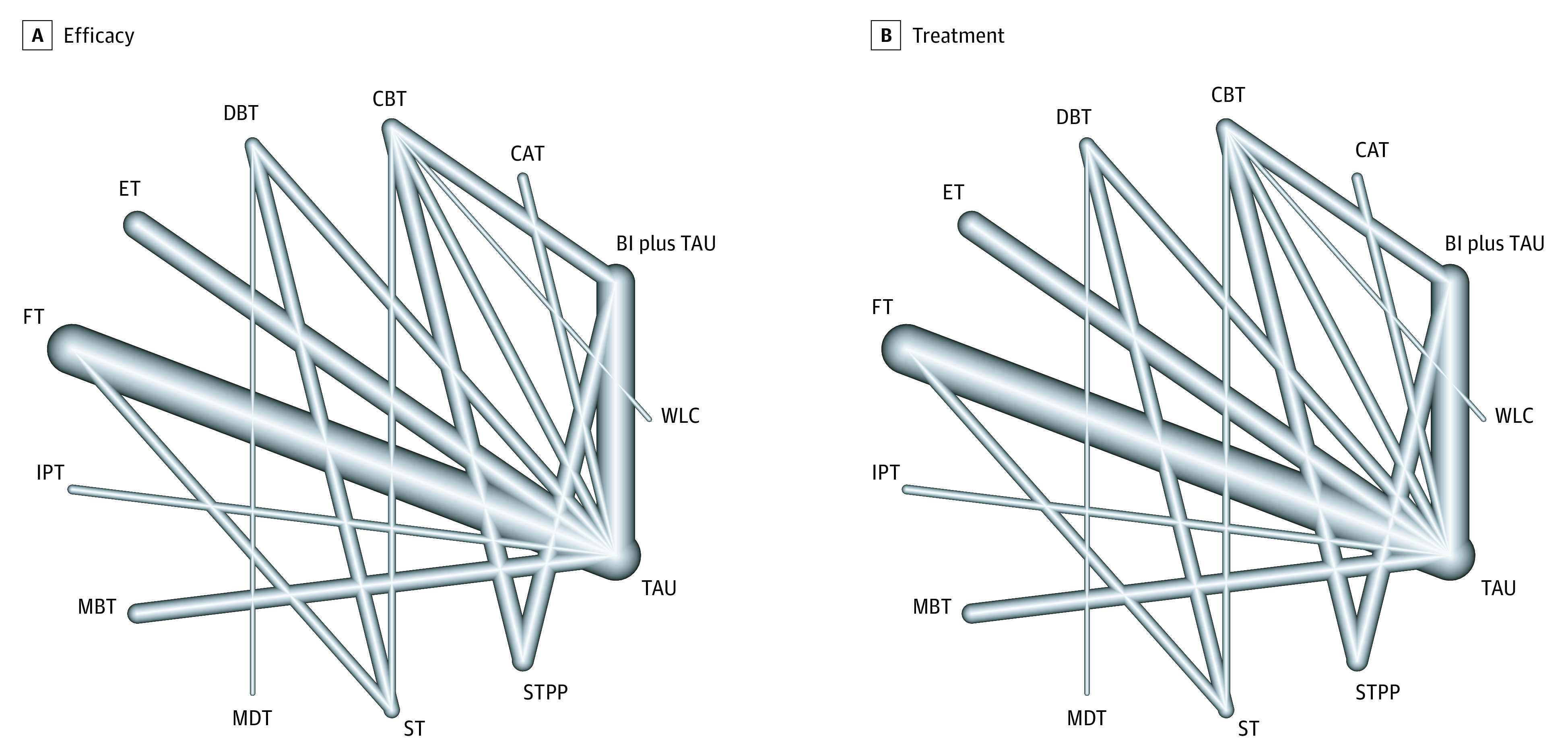
Network Plot of Eligible Psychotherapy Comparisons for Retention in Treatment Line width corresponds with the number of clinical trials comparing psychotherapy pairs. BI indicates brief intervention; CAT, cognitive analytic therapy; CBT, cognitive behavioral therapy; DBT, dialectical behavioral therapy; ET, eclectic therapy; FT, family-based therapy; IPT, interpersonal therapy; MBT, mentalization-based therapy; MDT, mode deactivation therapy; ST, supportive therapy; STPP, short-term psychoanalytic psychotherapy; TAU, treatment as usual; and WLC, wait-list control group.

The RCTs included in our review^47,48,50,51,52,53,54,55,56,57,58,59,60,61,62,63,64,65,66,67,68,69,70,71,72,73,74,75,76,77,78,79,80,81,82,83,84,85,86,87,88,89,90,91,92,93,94,95^ spanned 1995 to 2020, with most studies conducted in the US (Table 1). With regard to clinical samples, 31 RCTs examined any patient who presented with self-harm behaviors, and 8 RCTs involved adolescents with BPD. The median duration of treatment and follow-up was 3 months (range, 0.25-12.00 months) and 12 months (range, 1-36 months), respectively. Among the 44 RCTs included, 33 studies offered individual psychotherapy, and the most common modalities were brief intervention, family-based therapy, and DBT (Figure 2).

**Table 1.** Characteristics of Randomized Clinical Trials Included in Network Meta-analysis

Source	Treatment group (No. of participants)	Country	Age range, y	Clinical group
Alavi et al,^56^ 2013	CBT (15) vs WLC (15)	Iran	12-18	Depression
Apsche et al,^57^ 2006	Group DBT (10) vs MDT (10)	US	15-18	Aggression and conduct
Asarnow et al,^58^ 2011	FT (89) vs EUC (92)	US	10-18	Transdiagnostic
Asarnow et al,^59^ 2017	FT (20) vs EUC (22)	US	12-18	Transdiagnostic
Beck et al,^52^ 2020 and Jørgensen et al,^47^ 2020	Group MBT (55) vs TAU (56)	Denmark	14-17	BPD
Britton et al,^60^ 2014	DBT (52) vs TAU (58)	US	11-12	Transdiagnostic
Chanen et al,^61^ 2008	CAT (44) vs GCC (42)	Australia	15-18	BPD
Cooney et al,^62^ 2010	DBT (15) vs TAU (15)	New Zealand	13-19	Transdiagnostic
Cotgrove et al,^63^ 1995	BI plus TAU (47) vs TAU (58)	UK	10-16	Transdiagnostic
Cottrell et al,^51^ 2020 and Cottrell et al,^53^ 2018	FT (415) vs TAU (417)	UK	11-17	Transdiagnostic
Diamond et al,^64^ 2010	FT (35) vs EUC (31)	UK	12-17	Transdiagnostic
Diamond et al,^65^ 2019	FT (66) vs ST (63)	US	12-18	Transdiagnostic
Donaldson et al,^66^ 2005	SBT (15) vs ST (16)	US	12-17	Transdiagnostic
Esposito-Smythers et al,^67^ 2011	CBT (20) vs EUC (20)	US	13-17	SUD
Esposito-Smythers et al,^68^ 2017	FB-CBT (41) vs AAU (40)	US	13-18	SUD
Gleeson et al,^69^ 2012	CAT (8) vs TAU (8)	Australia	15-25	BPD plus psychosis
Goodyer et al,^70^ 2017	CBT (155) vs STPP (157) vs BI plus TAU (158)	UK	11-17	Depression
Green et al,^71^ 2011	Group ET (183) vs EUC (183)	UK	12-17	Transdiagnostic
Griffiths et al,^72^ 2019	Group MBT (26) vs TAU (27)	UK	12-18	Transdiagnostic
Harrington et al,^73^ 1998	FT (85) vs TAU (77)	UK	10-16	Transdiagnostic
Hazell et al,^74^ 2009	Group ET (35) vs TAU (37)	Australia	12-16	Transdiagnostic
Hetrick et al,^75^ 2017	CBT (26) vs TAU (24)	Australia	13-19	Transdiagnostic
Hill et al,^76^ 2019	IPT (41) vs TAU (39)	US	13-19	Transdiagnostic
Kaess et al,^77^ 2020	CBT plus DBT (37) vs TAU (37)	Multicenter	12-17	Transdiagnostic
Kennard et al,^78^ 2018	BI plus TAU (34) vs TAU (32)	US	12-18	Transdiagnostic
King et al,^79^ 2006	BI plus TAU (151) vs TAU (138)	US	12-17	Transdiagnostic
King et al,^80^ 2009	BI plus TAU (223) vs TAU (225)	US	13-17	Transdiagnostic
King et al,^81^ 2015	MI (27) vs EUC (22)	US	14-19	Transdiagnostic
McCauley et al,^82^ 2018	Group DBT (86) vs ST (87)	US	12-18	BPD
Mehlum et al,^48^ 2016 and Mehlum et al,^54^ 2014	Group DBT (39) vs EUC (38)	Norway	12-18	BPD
Ougrin et al,^50^ 2013 and Ougrin et al^55^ 2011	BI plus TAU (35) vs TAU (35)	Norway	12-18	Transdiagnostic
Ougrin et al,^83^ 2018	BI plus TAU (53) vs TAU (53)	UK	10-18	Transdiagnostic
Pineda et al,^84^ 2013	FT (24) vs TAU (24)	Australia	12-17	Transdiagnostic
Robinson et al,^85^ 2012	BI plus TAU (81) vs TAU (83)	Australia	15-24	Transdiagnostic
Rossouw et al,^86^ 2012	MBT (40) vs TAU (40)	UK	13-18	BPD
Santamarina-Perez et al,^87^ 2020	DBT (18) vs TAU (17)	Spain	12-17	Transdiagnostic
Schuppert et al,^88^ 2009	Group DBT (23) vs TAU (20)	Netherlands	14-19	BPD
Schuppert et al,^89^ 2012	Group DBT (54) vs TAU (55)	Netherlands	14-19	BPD
Sinyor et al,^90^ 2020	CBT (12) vs ST (12)	Canada	16-26	Transdiagnostic
Tang et al,^91^ 2009	IPT (35) vs TAU (38)	Taiwan	12-18	Transdiagnostic
Van Voorhees et al,^92^ 2009	MI (42) vs TAU (40)	US	14-21	Transdiagnostic
Wharff et al,^93^ 2019	FT (68) vs TAU (71)	US	13-18	Transdiagnostic
Wood et al,^94^ 2001	Group ET (32) vs TAU (37)	UK	12-16	Transdiagnostic
Yen et al,^95^ 2019	FT (27) vs TAU (27)	US	12-18	Transdiagnostic

Abbreviations: BI, brief intervention; BPD, borderline personality disorder; CAT, cognitive analytic therapy; CBT, cognitive behavioral therapy; DBT, dialectical behavioral therapy; ET, eclectic therapy; EUC, enhanced usual care; FT, family-based therapy; IPT, interpersonal therapy; MBT, mentalization-based therapy; MDT, mode deactivation therapy; ST, supportive therapy; STPP, short-term psychoanalytic psychotherapy; TAU, treatment as usual; UK, United Kingdom; WLC, wait-list control group.

### Risk of Bias

With regard to risk of bias within studies, most of the 44 RCTs reported adequate randomization (39 studies), adequate allocation concealment (33 studies), and blinded outcome assessors (36 studies). Only 27 RCTs were preregistered, and only 13 RCTs provided published protocols; 31 studies therefore had a high risk of bias for selective reporting. The risk of incomplete outcome reporting was increased in 11 RCTs because of insufficient details on attrition. Most RCTs reported information on funding (41 studies) and therapist adherence or fidelity (28 studies). However, a high risk of allegiance bias was found in 38 RCTs, and a high risk of attention bias was found in at least 10 RCTs (the risk of attention bias was unclear in an additional 27 studies). As a consequence, a high overall risk of bias was present in all 44 RCTs (eTable 3 in the Supplement).

To evaluate risk of bias across studies, we downgraded the quality of evidence for all outcomes owing to the high risk of bias in all included RCTs. We also downgraded the overall quality of evidence because of high heterogeneity in suicidal ideation and mood symptoms and imprecision for psychotherapies that had few representative RCTs (eg, mode deactivation therapy and short-term psychoanalytic psychotherapy had only 1 representative RCT each). Although inconsistency was low, publication bias was found for self-harm frequency at the end of treatment (Table 2).

**Table 2.** Network Meta-analysis Indices

Variable	No. of studies	No. of treatments	No. of pairwise comparisons	τ^2^^a^	*I*^2^, %^b^	*Q* between^c^	*P* value for *Q* between^d^	Egger *P* value
Retention in treatment	44	13	46	0.15	32.6	3.06	.38	.21
Study withdrawals	44	13	46	0.16	33.7	2.95	.40	.23
End of treatment								
Self-harm	16	10	18	0.09	14.5	5.66	.06	.01
Suicidal ideation	31	12	31	0.17	81.8	0.65	.72	.24
Mood	28	13	30	0.12	75.6	3.59	.17	.12
Follow-up								
Self-harm	25	10	27	<0.01	1.2	4.40	.11	.07
Suicidal ideation	19	11	19	0.63	94.9	0.07	.80	.53
Mood	19	12	21	0.06	71.4	<0.01	.97	.05

^a^ Heterogeneity between designs.

^b^ Heterogeneity within designs.

^c^ Inconsistency between designs.

^d^ Significance of heterogeneity for *Q*-between statistic.

### Synthesis of Findings

**Figure 3.**
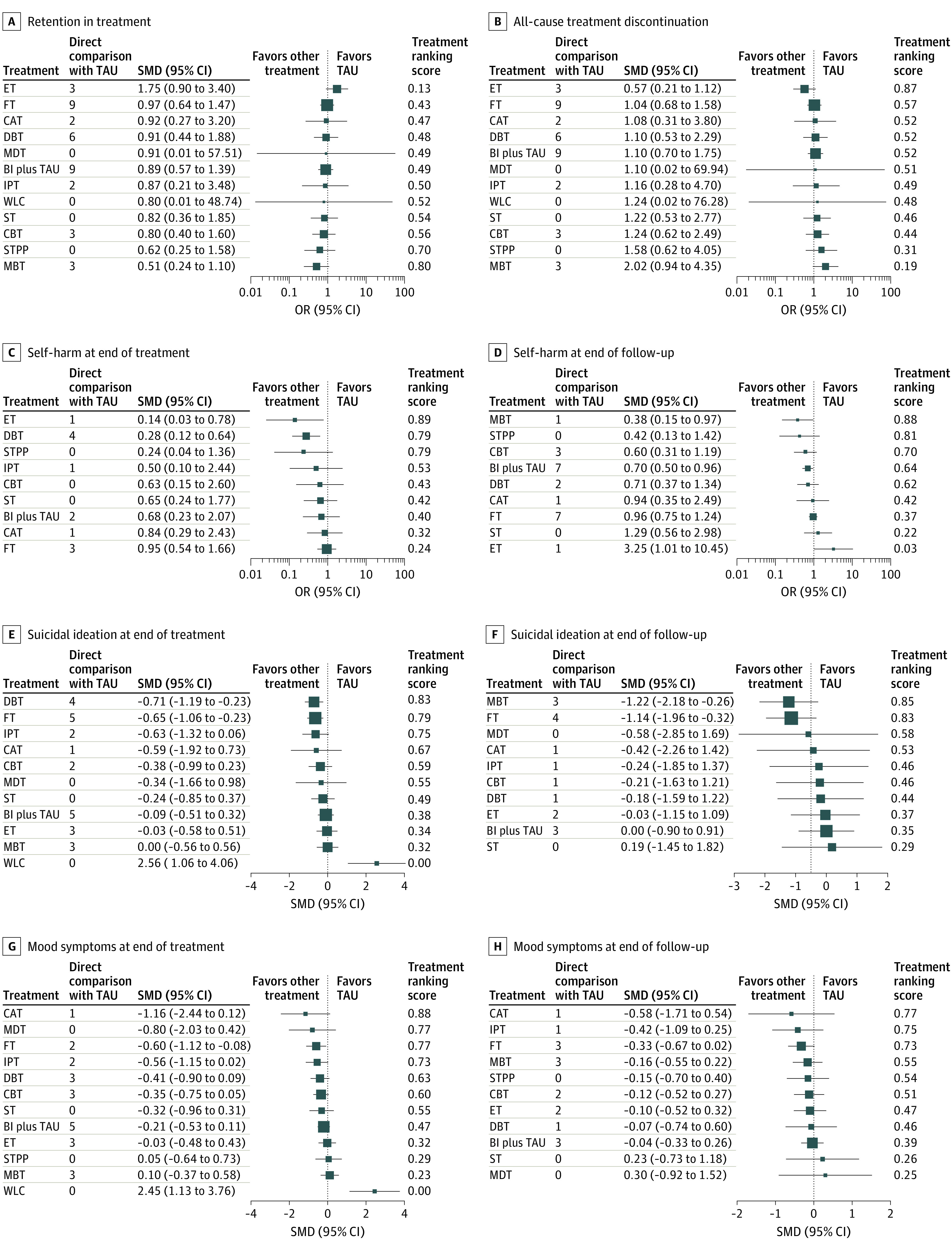
Forest Plots of Treatment Acceptability Across All Clinical Trials in Network Meta-analysis All psychotherapies were compared with treatment as usual (TAU) using a random-effects model. For treatment ranking score, treatments at the top of the plots have higher ranking. OR indicates odds ratio; SMD, Cohen *d* standardized mean difference. All other definitions appear in the Figure 2 caption.

None of the investigated psychotherapies were associated with more study withdrawals compared with treatment as usual (Figure 3A and Figure 3B). However, efficacy was inconsistent across outcomes and psychotherapies. For example, eclectic therapy and DBT were associated with reductions in self-harm at the end of treatment (OR, 0.14 [95% CI, 0.03-0.78] for eclectic therapy and 0.28 [95% CI, 0.12-0.64] for DBT) (Figure 3C), while DBT and family-based therapy were associated with reductions in suicidal ideation at the end of treatment (Cohen *d* SMD, −0.71 [95% CI, −1.19 to −0.23] for DBT and −0.65 [95% CI, −1.06 to −0.23] for family-based therapy) compared with treatment as usual (Figure 3E). For depressive symptoms, only family-based therapy was associated with reductions in symptom severity at the end of treatment (Cohen *d* SMD, −0.60; 95% CI, −1.12 to −0.08) (Figure 3G).

In extended follow-up, only MBT and brief intervention plus treatment as usual were associated with decreases in self-harm (OR, 0.38 [95% CI, 0.15-0.97] for MBT and 0.70 [95% CI, 0.50-0.96] for brief intervention plus treatment as usual) (Figure 3D), and only MBT and family-based therapy were associated with reductions in suicidal ideation (Cohen *d* SMD, −1.22 [95% CI, −2.18 to −0.26] for MBT and −1.14 [95% CI, −1.96 to −0.32] for family-based therapy) compared with treatment as usual (Figure 3F). None of the investigated therapies were associated with improvements in depressive symptoms over longer follow-up periods (Figure 3H). Participants in the wait-list control groups experienced worsening conditions, with increases in self-harm, mood symptoms, and suicidal ideation compared with participants receiving treatment as usual.

## Discussion

Although the present NMA found that most psychotherapies were reasonably well tolerated and some psychotherapies indicated efficacy for particular measures of self-harm or suicidality, caution is recommended to avoid overinterpretation of these findings owing to low RCT quality, lack of consistency across outcome measures and treatment periods, and publication bias. When significant, most psychotherapies had small to medium effects compared with treatment as usual. Substantial reductions in self-harm and suicidal behavior were often observed in both the treatment and control groups, and group differences were subsequently small and nonsignificant for many RCTs.

The present NMA is not the first, and is unlikely to be the last, study to review psychotherapeutic efficacy for self-harm and suicidality among children and adolescents.^8,9,15,16,96,97,98,99,100,101,102,103^ Although the present review did not focus on a specific clinical sample, relevant insights can be drawn from studies of adolescents with particular diagnoses, such as BPD. For example, Wong et al^104^ reported that a range of psychotherapies, including DBT and MBT, were associated with short-term, but not long-term, reductions in BPD symptomatology. However, as in previous reviews, Wong et al^104^ observed diminishing therapeutic efficacy over time, as psychotherapy effect sizes decreased during follow-up relative to the end of treatment. In addition, the clinical trials included in the review by Wong et al^104^ were of varying lengths and reported variable outcome measures for different dimensions of BPD symptomatology and functioning, which introduced several limitations in the formulation of firmer conclusions about the relative benefits of other therapies. In the present NMA, decreasing efficacy during follow-up compared with the end of treatment was also observed. Although it was more challenging to directly assess this pattern in the present NMA because of the varying numbers of studies reporting data on end of treatment and follow-up for particular psychotherapies, this challenge is not unique to our review.

Most previous meta-analyses of psychotherapies for children and adolescents with suicidal behaviors have identified similar limitations, highlighting the need for additional research and large-scale RCTs.^2^ Conducting research on self-harm and suicidal behavior among adolescents is intrinsically challenging because of the distinct trajectory of self-harm, the transient nature of some suicidal behaviors, and the nature of control interventions, which can often confer therapeutic benefits.^2^ Despite these challenges, the present review does not intend to downgrade the overall utility of psychotherapies, which remain useful for the treatment of a range of mental disorders, often as first-line interventions. However, the diverse array of psychotherapies and their evaluation in individual RCTs produced methodological challenges in creating a clear hierarchy of treatment rankings, which was the intended aim of this review. In part, the most challenging aspect of this review was synthesizing the data across a range of diverse RCTs that explored different psychotherapeutic modalities. Thus, the high risk of bias in the individual RCTs of psychotherapies for self-harm and suicidality among children and adolescents may have had implications for the findings.

Several approaches have emerged in studies of child and adolescent psychiatry that may support future comparative effectiveness research involving psychotherapies for self-harm. For example, a 2021 review by Jørgensen et al^18^ extended previous meta-analyses of BPD studies by conducting a trial sequential analysis, which aids the interpretation of meta-analyses involving sparse data and helps to address type 1 and type 2 errors. An alternative approach involves individual participant-level analyses and comprises pooling individual-level data to arrive at a single estimate of a treatment’s efficacy rather than a summary of aggregate RCT-level estimates. As a consequence, using data from large longer-term observational studies, such as phase 4 clinical trials, could be another option, which may also provide more real-world estimates of treatment effectiveness rather than efficacy.^105^

### Strengths and Limitations

This study has strengths. To our knowledge, this review is the first to apply NMA to evaluate psychotherapies for the treatment of self-harm and suicidality among children and adolescents. Given the abundance of single-treatment RCTs and the shortage of head-to-head RCTs, the use of NMAs can provide a novel approach to synthesizing knowledge with the data available.^106,107^

This study also has several limitations. Although NMA is a powerful tool for comparative effectiveness research, it can produce misleading results when misapplied or misinterpreted. Most of our evidence relied on indirect treatment comparisons; when using head-to-head comparisons, indirect observations are more susceptible to bias. For a subset of psychotherapies (eg, mode deactivation therapy, short-term psychoanalytic psychotherapy, and supportive therapy), the availability of few RCTs and the use of small samples creates imprecise and potentially underpowered estimates. Although we pooled studies regardless of diagnostic classification to maximize statistical power, the findings of this review are less generalizable to specific clinical populations, such as adolescents with BPD.^108,109^ As a consequence, high heterogeneity was observed in some outcomes; however, given the lack of standardized protocols for RCTs investigating psychotherapy, this heterogeneity was, to a certain extent, unavoidable and not a specific limitation of this review.^110^ Although the RCTs examining family-based therapy were similar, the number of sessions ranged from 1 to 12; this difference may have produced additional heterogeneity. The duration of psychotherapy is another possible source of heterogeneity. We used the random-effects model to estimate effect sizes across different instruments measuring suicidality or depression, and we assumed that these instruments measured the same construct. However, this assumption was not definitively assessed and could have increased heterogeneity.

In addition to the challenges inherent in blinded clinical trials of psychotherapies,^111^ the risk of bias in individual RCTs was high because of other factors, particularly allegiance,^112^ selective reporting, and incomplete outcome reporting biases. Response and social desirability biases could have produced biased self-reported subjective measures, to which self-harm and suicidal ideation are particularly susceptible.^113^ Despite an extensive search, we may have missed relevant RCTs, given the publication bias in one of our primary outcomes. Although we did not detect network-level publication bias for most other outcomes, individual psychotherapies may have been subject to publication bias, as only 1 study was conducted for some interventions (eg, mode deactivation therapy and short-term psychoanalytic psychotherapy). Therapy-comparator differences could have been diminished by the active therapeutic nature of some comparator conditions, such as treatment as usual and enhanced usual care, which often provide unstructured psychotherapy sessions independent of a particular psychological modality.^2^

## Conclusions

Although the findings of this review suggest that some psychotherapies are well tolerated and have some efficacy for specific measures of self-harm or suicidality, the estimates indicated that the evidence quality was low to very low for most psychotherapies. A lack of consistent evidence precludes a definitive hierarchy of treatments and suggests a need for additional high-quality RCTs.

## Notes

Supplement. **eMethods.** Network Meta-analysis Code **eTable 1.** Psychotherapy Definitions **eTable 2.** Search Strategy **eTable 3.** Risk of Bias **eReferences**
