# Supplementary material for: Comparative Efficacy and Acceptability of Psychotherapies for Self-harm and Suicidal Behavior Among Children and Adolescents: A Systematic Review and Network Meta-analysis
Source: JAMA Netw Open. 2021 Apr 16;4(4):e216614. doi: 10.1001/jamanetworkopen.2021.6614 (PMC8052594; doi:10.1001/jamanetworkopen.2021.6614)
Supplement: Supplement. — eMethods. Network Meta-analysis Code eTable 1. Psychotherapy Definitions eTable 2. Search Strategy eTable 3. Risk of Bias eReferences [file jamanetwopen-e216614-s001.pdf]

# Supplementary Online Content

Bahji A, Pierce M, Wong J, Roberge JN, Ortega I, Patten S. Comparative efficacy and acceptability of psychotherapies for self-harm and suicidal behavior among children and adolescents: a systematic review and network meta-analysis. *JAMA Netw Open*. 2021;4(4):e216614. doi:10.1001/jamanetworkopen.2021.6614

**eMethods.** Network Meta-analysis Code

**eTable 1.** Psychotherapy Definitions

**eTable 2.** Search Strategy

**eTable 3.** Risk of Bias

**eReferences**

This supplementary material has been provided by the authors to give readers additional information about their work.

© 2021 Bahji A et al. *JAMA Network Open*.

**eMethods.** Network Meta-analysis Code

```
library(readxl)
library(netmeta)
library(meta)
library(tidyverse)
data <- read_excel("~/File Location/C&A.xlsx", sheet = "Main")
attach(data)

################################################################################
######
# demographics

sum(N)
mean(Prop.F, na.rm = TRUE)
table(Year)
table(Country)
table(`Age Group`) %>% sort(decreasing = TRUE)
table(Clinical)

median(EOT_Months)
median(FU_Months, na.rm = TRUE)
table(Modality)
table(c(T1.class,T2.class,T3.class)) %>% sort(decreasing = TRUE)

################################################################################
######
#risk of bias
table(Randomization)
table(Allocation)
table(Blinding)
table(Registered)
table(Protocol)
table(`Selective Reporting`)
table(`Incomplete (Attrition)`)
table(Funding)
table(Adherence)
table(Allegiance)
table(Attention)
table(Overall)

################################################################################
######
## Outcome 1: retention in treatment
p1 <- pairwise(list(T1.class, T2.class, T3.class),
        event = list(R1, R2, R3),
        n = list(N1, N2, N3),
```

© 2021 Bahji A et al. *JAMA Network Open*.

```
              studlab = Study,
              data = data,
              sm = "OR",
              allstudies = TRUE)
net1 <- netmeta(p1, comb.fixed = FALSE, reference = "TAU")
print(summary(net1), digits = 2)
decomp.design(net1)
netgraph(net1, seq = net1$trts)
forest(net1, ref="TAU", xlab="Odds ratio for retention in treatment",
       rightcols=c("effect", "ci", "Pscore"), rightlabs="P-Score",
       leftcols=c("studlab", "k"),
       leftlabs=c("Contrast\nto TAU", "Direct\nComparisons"),
       just.addcols="right",
       sortvar=Pscore,
       smlab = "Random Effects Model",
       drop.reference.group = TRUE)
trts_1 <- c(net1$trts)
funnel(net1, order = trts_1, linreg = TRUE,
       digits.pval = 2, legend = FALSE, col = "red",
       xlab = "Odds ratio for retention in treatment")

################################################################################
######
## Outcome 2: dropouts
p2 <- pairwise(list(T1.class, T2.class, T3.class),
              event = list(D1, D2, D3),
              n = list(N1, N2, N3),
              studlab = Study,
              data = data,
              sm = "OR",
              allstudies = TRUE)
net2 <- netmeta(p2, comb.fixed = FALSE, reference = "TAU")
print(summary(net2), digits = 2)
decomp.design(net2)
netgraph(net2)
forest(net2, ref="TAU", xlab="Odds ratio for dropouts",
       rightcols=c("effect", "ci", "Pscore"),
       rightlabs="P-Score",
       leftcols=c("studlab", "k"),
       leftlabs=c("Contrast\nto TAU", "Direct\nComparisons"),
       just.addcols="right",
       sortvar=-Pscore,
       smlab = "Random Effects Model",
       drop.reference.group = TRUE)
trts_2 <- c(net2$trts)
funnel(net2, order = trts_2, linreg = TRUE,
```

© 2021 Bahji A et al. *JAMA Network Open*.

```
      digits.pval = 2, legend = FALSE, col = "purple",
      xlab = "Odds ratio for dropouts")

################################################################################
######
## Outcome 3: self-harm @ EOT
p3 <- pairwise(list(T1.class, T2.class, T3.class),
           event = list(SH1_EOT, SH2_EOT, SH3_EOT),
           n = list(N1, N2, N3),
           studlab = Study,
           data = data,
           sm = "OR",
           allstudies = TRUE)
net3 <- netmeta(p3, comb.fixed = FALSE, reference = "TAU")
print(summary(net3), digits = 2)
decomp.design(net3)
netgraph(net3, seq = net3$trts)
forest(net3, ref="TAU", xlab="Odds ratio for self harm reduction @ EOT",
      rightcols=c("effect", "ci", "Pscore"),
      rightlabs="P-Score",
      leftcols=c("studlab", "k"),
      leftlabs=c("Contrast\nto TAU", "Direct\nComparisons"),
      just.addcols="right",
      sortvar=-Pscore,
      smlab = "Random Effects Model",
      drop.reference.group = TRUE)
trts_3 <- c(net3$trts)
funnel(net3, order = trts_3, linreg = TRUE,
      digits.pval = 2, legend = FALSE, col = "orange",
      xlab = "Odds ratio for self harm reduction @ EOT")

################################################################################
######
## Outcome: SI @ EOT
p4 <- pairwise(list(T1.class, T2.class, T3.class),
           mean = list(SI1.m_EOT, SI2.m_EOT, SI3.m_EOT),
           sd = list(SI1.sd_EOT, SI2.sd_EOT, SI3.sd_EOT),
           n = list(N1, N2, N3),
           studlab = Study,
           data = data,
           sm = "SMD",
           allstudies = TRUE)
net4 <- netmeta(p4, comb.fixed = FALSE, reference = "TAU")
print(summary(net4), digits = 2)
decomp.design(net4)
netgraph(net4)
```

© 2021 Bahji A et al. *JAMA Network Open*.

```
forest(net4, ref="TAU", xlab="Standardized mean difference for suicidal ideation @ EOT",
      rightcols=c("effect", "ci", "Pscore"), rightlabs="P-Score",
      leftcols=c("studlab", "k"),
      leftlabs=c("Contrast\nto TAU", "Direct\nComparisons"),
      just.addcols="right",
      sortvar=-Pscore,
      smlab = "Random Effects Model",
      drop.reference.group = TRUE)
trts_4 <- c(net4$trts)
funnel(net4, order = trts_4, linreg = TRUE,
      digits.pval = 2, legend = FALSE, col = "green",
      xlab = "Standardized mean difference for suicidal ideation @ EOT")

##########################################################################################
######
## Outcome: mood symptoms @ EOT
p5 <- pairwise(list(T1.class, T2.class, T3.class),
            mean = list(MDD1.m_EOT, MDD2.m_EOT, MDD3.m_EOT),
            sd = list(MDD1.sd_EOT, MDD2.sd_EOT, MDD3.sd_EOT),
            n = list(N1, N2, N3),
            studlab = Study,
            data = data,
            sm = "SMD")
net5 <- netmeta(p5, comb.fixed = FALSE, reference = "TAU")
print(summary(net5), digits = 2)
decomp.design(net5)
netgraph(net5)
forest(net5, ref="TAU", xlab="Standardized mean difference for mood symptoms @ EOT)",
      rightcols=c("effect", "ci", "Pscore"), rightlabs="P-Score",
      leftcols=c("studlab", "k"),
      leftlabs=c("Contrast\nto TAU", "Direct\nComparisons"),
      just.addcols="right",
      sortvar=-Pscore,
      smlab = "Random Effects Model",
      drop.reference.group = TRUE)
trts_5 <- c(net5$trts)
funnel(net5, order = trts_5, linreg = TRUE,
      digits.pval = 2, legend = FALSE, col = "blue",
      xlab = "Standardized mean difference for mood symptoms @ EOT")


##########################################################################################
######
## Outcome 7: self-harm @ FU
p7 <- pairwise(list(T1.class, T2.class, T3.class),
            event = list(SH1_FU, SH2_FU, SH3_FU),
```

© 2021 Bahji A et al. *JAMA Network Open*.

```
            n = list(N1, N2, N3),
            studlab = Study,
            data = data,
            sm = "OR")
net7 <- netmeta(p7, comb.fixed = FALSE, reference = "TAU")
print(summary(net7), digits = 2)
decomp.design(net7)
netgraph(net7)
forest(net7, ref="TAU", xlab="Odds ratio for self harm reduction @ FU",
      rightcols=c("effect", "ci", "Pscore"),
      rightlabs="P-Score",
      leftcols=c("studlab", "k"),
      leftlabs=c("Contrast\nto TAU", "Direct\nComparisons"),
      just.addcols="right",
      sortvar=-Pscore,
      smlab = "Random Effects Model",
      drop.reference.group = TRUE)
trts_7 <- c(net7$trts)
funnel(net7, order = trts_7, linreg = TRUE,
      digits.pval = 2, legend = FALSE, col = "black",
      xlab = "Odds ratio for self harm reduction @ FU")

##################################################################################
######
## Outcome: SI @ FU
p8 <- pairwise(list(T1.class, T2.class, T3.class),
            mean = list(SI1.m_FU, SI2.m_FU, SI3.m_FU),
            sd = list(SI1.sd_FU, SI2.sd_FU, SI3.sd_FU),
            n = list(N1, N2, N3),
            studlab = Study,
            data = data,
            sm = "SMD")
net8 <- netmeta(p8, comb.fixed = FALSE, reference = "TAU")
print(summary(net8), digits = 2)
decomp.design(net8)
netgraph(net8)
forest(net8, ref="TAU", xlab="Standardized mean difference for suicidal ideation @ FU",
      rightcols=c("effect", "ci", "Pscore"), rightlabs="P-Score",
      leftcols=c("studlab", "k"),
      leftlabs=c("Contrast\nto TAU", "Direct\nComparisons"),
      just.addcols="right",
      sortvar=-Pscore,
      smlab = "Random Effects Model",
      drop.reference.group = TRUE)
trts_8 <- c(net8$trts)
funnel(net8, order = trts_8, linreg = TRUE,
```

© 2021 Bahji A et al. *JAMA Network Open*.

```
      digits.pval = 2, legend = FALSE, col = "red",
      xlab = "Standardized mean difference for suicidal ideation @ FU")

##############################################################################
######
## Outcome: mood symptoms (continous) @ FU
p9 <- pairwise(list(T1.class, T2.class, T3.class),
            mean = list(MDD1.m_FU, MDD2.m_FU, MDD3.m_FU),
            sd = list(MDD1.sd_FU, MDD2.sd_FU, MDD3.sd_FU),
            n = list(N1, N2, N3),
            studlab = Study,
            data = data,
            sm = "SMD")
net9 <- netmeta(p9, comb.fixed = FALSE, reference = "TAU")
print(summary(net9), digits = 2)
decomp.design(net9)
netgraph(net9)
forest(net9, ref="TAU", xlab="Standardized mean difference for mood symptoms @ FU",
      rightcols=c("effect", "ci", "Pscore"), rightlabs="P-Score",
      leftcols=c("studlab", "k"),
      leftlabs=c("Contrast\nto TAU", "Direct\nComparisons"),
      just.addcols="right",
      sortvar=-Pscore,
      smlab = "Random Effects Model",
      drop.reference.group = TRUE)
trts_9 <- c(net9$trts)
funnel(net9, order = trts_9, linreg = TRUE,
      digits.pval = 2, legend = FALSE, col = "green",
      xlab = "Standardized mean difference for mood symptoms @ FU")
```

© 2021 Bahji A et al. *JAMA Network Open*.

**eTable 1.** Psychotherapy Definitions

| | |
|---|---|
| *Brief Intervention (BI)* | A multifaceted intervention including psychoeducation, support for increased activities, and attention to the person's family and school environment.[1] BI involves low resource, non-intrusive interventions that aim to facilitate an ongoing therapeutic relationship with patients. No additional therapy is given and has mostly been used with clinical populations following presentation to an emergency department.[2] |
| *Cognitive Analytic Therapy (CAT)* | A short form of talk therapy allows people to understand their current difficulties and develop new coping methods. |
| *Cognitive Behavioural Therapy (CBT)* | Uses techniques such as restructuring, regulation of affect, development of skills to improve communication and maladaptive cognitions.[3,4] |
| *Dialectical Behavioural Therapy (DBT)* | DBT can address cognitive-behavioural and regulatory processes such as distress tolerance and emotion regulation and managing social situations.[5,6] May also involve mindfulness, defined as the practice of attention regulation and emotional stability.[7] |
| *Eclectic Therapy (ET)* | An open, integrative form of psychotherapy that adapts to the unique needs of each specific client, depending on the problem, the treatment goals, and the person's expectations and motivation.[8] |
| *Family Therapy (FT)* | Builds a collaborative way of communicating between adolescents and parents. It may identify the causes, reactions, and future response to suicidal behaviours through psychoeducation, cognitive-behavioural skill-building, and safety plans development.[9–11] |
| *Interpersonal Therapy (IPT)* | Target symptoms are identified and are linked to interpersonal problems. This concept can also be used to treat symptoms involving suicide and depression.[12] |
| *Mentalization-Based Therapy (MBT)* | Utilizes the concepts of attachment theory. It requires motivation from the patient and focuses on impulsivity, affecting regulation building the patients' capacity to understand and demonstrate feelings in challenging situations.[13,14] Uses an integrated model of development and psychopathology to foster the development of coping skills to manage stressful situations that may result in repeating pathological behaviours.[1] |
| *Mode Deactivation Therapy (MDT)* | MDT is a systematic, manualized, contextualized therapy embedding elements from CBT, ACT, DBT, and the validation-clarification-redirection change technique. |
| *Supportive Therapy (ST)* | ST emphasizes therapeutic alliance to alleviate symptoms, improve self-esteem, regulate impulses, and reinforce coping skills. |
| *Short-Term Psychoanalytic Psychotherapy (STPP)* | STPP was developed as a brief alternative to the original long-term models. There have been several variants developed since the 1970s. Still, all are brief talking therapies aimed to work with unconscious impulses, feelings and processes that can cause or maintain chronic mental disorders. |
| *Treatment as Usual (TAU)* | TAU includes a range of treatment techniques and modalities, such as supportive counselling or cognitive behaviour therapy. It is diverse and |

© 2021 Bahji A et al. *JAMA Network Open*.

| | |
|---|---|
| | may involve individual and/or family work, delivered by a range of practitioners with various theoretical backgrounds.[9,15] |

© 2021 Bahji A et al. *JAMA Network Open*.

**eTable 2.** Search Strategy

**PubMed:** inception to September 15, 2020

| Step | Search Criteria | Citations |
|---|---|---|
| 1. | ("Self-harm" OR "Self-injury" OR "Suicidal behaviour" OR "Suicidal ideation" OR "Cutting" OR "Suicide") AND ("Adolescen*") AND ("Therapy" OR "Intervention") | 6676 |
| 2. | Limit 1 to clinical trial, human | 649 |

**MEDLINE:** inception to September 15, 2020

| Step | Search Criteria | Citations |
|---|---|---|
| 1. | exp Suicide/ or exp Suicide, Completed/ or exp Suicide, Attempted/ or suicide.mp. | 86862 |
| 2. | exp Self-Injurious Behavior/ or self harm.mp. | 73517 |
| 3. | exp Adolescent/ or adolescent.mp. | 2069566 |
| 4. | exp Psychotherapy, Psychodynamic/ or exp Psychotherapy, Group/ or exp Psychotherapy, Rational-Emotive/ or psychotherapy.mp. or exp Psychotherapy, Brief/ or exp Psychotherapy, Multiple/ or exp Interpersonal Psychotherapy/ or exp Psychotherapy/ or exp Imagery, Psychotherapy/ or exp Person-Centered Psychotherapy/ | 206025 |
| 5. | therapy.mp. or exp Therapeutics/ | 7525771 |
| 6. | (1 or 2) and 3 and (4 or 5) | 7243 |
| 7. | Limit 6 to randomized controlled trial, human | 367 |

**PsycINFO:** inception to September 15, 2020

| Step | Search Criteria | Citations |
|---|---|---|
| 1. | exp Attempted Suicide/ or exp Suicide Prevention/ or exp Suicide/ or suicide.mp. | 58439 |
| 2. | exp Self-Injurious Behavior/ or self harm.mp. | 10028 |
| 3. | adolescent.mp. | 382861 |
| 4. | psychotherapy.mp. or exp Psychotherapy/ | 245790 |
| 5. | therapy.mp. or exp Treatment/ | 1106871 |
| 6. | (1 or 2) and 3 and (4 or 5) | 4727 |

© 2021 Bahji A et al. *JAMA Network Open*.

| | | |
|---|---|---|
| 7. | Limit 6 to randomized controlled trial, human | 166 |

**EMBASE:** inception to September 15, 2020

| Step | Search Criteria | Citations |
|---|---|---|
| 1. | exp suicide/ or suicide.mp. or exp suicide attempt/ | 108339 |
| 2. | self harm.mp. or exp automutilation/ | 21049 |
| 3. | exp adolescent/ or adolescent.mp. | 1569623 |
| 4. | therapy.mp. or exp therapy/ | 11157451 |
| 5. | psychotherapy.mp. or exp interpersonal psychotherapy/ or exp psychotherapy/ or exp short term psychotherapy/ or exp psychodynamic psychotherapy/ | 258201 |
| 6. | 3 and 6 and 7 | 6861 |
| 7. | Limit 6 to randomized controlled trial, human | 363 |

© 2021 Bahji A et al. *JAMA Network Open*.

**eTable 3.** Risk of Bias

| Study | Randomization | Allocation | Blinding | Registered | Protocol | Selective Reporting | Incomplete (Attrition) | Funding | Adherence | Allegiance | Attention | Overall |
|---|---|---|---|---|---|---|---|---|---|---|---|---|
| **Alavi et al., 2013**[16] | High risk | High risk | Unclear risk | Unclear | Unclear | Unclear risk | Low risk | Low risk | Unclear risk | Low risk | High risk | High risk |
| **Apsche et al., 2006**[17] | High risk | High risk | Unclear risk | Unclear | Unclear | Unclear risk | Low risk | Low risk | Unclear risk | High risk | Unclear risk | High risk |
| **Asarnow et al., 2011**[18] | Low risk | Low risk | Low risk | Registered | Unclear | High risk | Low risk | Low risk | Unclear risk | Low risk | Unclear risk | High risk |
| **Asarnow et al., 2017**[19] | Low risk | Low risk | Low risk | Registered | Unclear | Low risk | Low risk | Low risk | Low risk | High risk | Low risk | High risk |
| **Beck et al., 2020**[20,21] | Low risk | Low risk | Low risk | Registered | Published | Low risk | Unclear risk | Low risk | Low risk | High risk | High risk | High risk |
| **Britton et al., 2014**[7] | Low risk | High risk | Low risk | Unclear | Unclear | High risk | Low risk | Low risk | Unclear risk | Low risk | Low risk | High risk |
| **Chanen et al., 2008**[22] | Low risk | Low risk | Low risk | Unclear | Unclear | Unclear risk | Unclear risk | Low risk | Low risk | High risk | Low risk | High risk |
| **Cooney et al., 2010**[23] | Low risk | Low risk | Low risk | Unclear | Published | High risk | Low risk | High risk | Low risk | High risk | Low risk | High risk |
| **Cotgrove et al., 1995**[24] | High risk | High risk | High risk | Unclear | Unclear | High risk | Low risk | High risk | Unclear risk | Unclear risk | Unclear risk | High risk |
| **Cottrell et al., 2018**[9,25] | Low risk | Low risk | Low risk | Registered | Published | Low risk | High risk | Low risk | Low risk | High risk | Unclear risk | High risk |
| **Diamond et al., 2010**[26] | Low risk | Low risk | High risk | Registered | Unclear | High risk | Low risk | Low risk | Low risk | High risk | Unclear risk | High risk |
| **Diamond et al., 2019**[27] | High risk | High risk | Low risk | Registered | Unclear | Low risk | Low risk | Low risk | Low risk | High risk | Unclear risk | High risk |
| **Donaldson et al., 2005**[28] | High risk | High risk | Unclear risk | Unclear | Unclear | High risk | Low risk | Low risk | Low risk | High risk | Unclear risk | High risk |
| **Esposito-Smythers et al., 2011**[29] | Low risk | High risk | High risk | Unclear | Unclear | High risk | Low risk | Low risk | Low risk | High risk | High risk | High risk |

© 2021 Bahji A et al. *JAMA Network Open*.

| | | | | | | | | | | | | |
|---|---|---|---|---|---|---|---|---|---|---|---|---|
| **Esposito-Smythers et al., 2017[3]** | Low risk | Low risk | Low risk | Registered | Unclear | High risk | High risk | Low risk | Low risk | High risk | High risk | High risk |
| **Gleeson et al., 2012[30]** | Low risk | High risk | High risk | Registered | Published | Low risk | High risk | High risk | Unclear risk | High risk | Low risk | High risk |
| **Goodyer et al., 2017[1]** | Low risk | Low risk | Low risk | Registered | Published | Low risk | Low risk | Low risk | Low risk | High risk | High risk | High risk |
| **Green et al., 2011[31]** | Low risk | Low risk | Low risk | Registered | Unclear | High risk | Low risk | Low risk | Low risk | High risk | Unclear risk | High risk |
| **Griffiths et al., 2019[13]** | Low risk | Low risk | Low risk | Registered | Published | Low risk | Low risk | Low risk | Low risk | High risk | Unclear risk | High risk |
| **Harrington et al., 1998[11]** | Low risk | Low risk | High risk | Unclear | Unclear | High risk | Low risk | Low risk | Unclear risk | High risk | Unclear risk | High risk |
| **Hazell et al., 2009[32]** | Low risk | Low risk | Low risk | Unclear | Unclear | High risk | High risk | Low risk | Low risk | High risk | Unclear risk | High risk |
| **Hetrick et al., 2017[4]** | Low risk | Low risk | Low risk | Unclear | Unclear | High risk | High risk | Low risk | Unclear risk | High risk | Unclear risk | High risk |
| **Hill and Pettit 2019[33]** | Low risk | Low risk | Low risk | Unclear | Unclear | High risk | Low risk | Low risk | Unclear risk | High risk | Unclear risk | High risk |
| **Kaess et al., 2020[34]** | Low risk | Low risk | Low risk | Registered | Published | High risk | Low risk | Low risk | Low risk | High risk | Unclear risk | High risk |
| **Kennard et al., 2018[35]** | Low risk | Low risk | Low risk | Registered | Unclear | Low risk | Low risk | Low risk | Low risk | High risk | Unclear risk | High risk |
| **King et al., 2006[36]** | Low risk | High risk | Low risk | Registered | Unclear | High risk | Low risk | Low risk | Low risk | High risk | Unclear risk | High risk |
| **King et al., 2009[37]** | Low risk | Low risk | Low risk | Registered | Unclear | High risk | Low risk | Low risk | Low risk | High risk | Unclear risk | High risk |
| **King et al., 2015[38]** | Low risk | Low risk | Low risk | Registered | Unclear | High risk | Low risk | Low risk | Low risk | High risk | Unclear risk | High risk |
| **McCauley et al., 2018[5]** | Low risk | Low risk | Low risk | Registered | Published | Low risk | Low risk | Low risk | Unclear risk | High risk | High risk | High risk |
| **Mehlum et al., 2014[6,39]** | Low risk | Low risk | Low risk | Registered | Published | Low risk | Low risk | Low risk | Unclear risk | High risk | High risk | High risk |

© 2021 Bahji A et al. *JAMA Network Open*.

| | | | | | | | | | | | | |
|---|---|---|---|---|---|---|---|---|---|---|---|---|
| **Ougrin et al., 2011[40,41]** | Low risk | Low risk | Low risk | Registered | Unclear | High risk | Low risk | Low risk | Low risk | High risk | Unclear risk | High risk |
| **Ougrin et al., 2018[15]** | Low risk | Low risk | Low risk | Registered | Unclear | Low risk | Low risk | Low risk | Unclear risk | High risk | Unclear risk | High risk |
| **Pineda et al., 2013[42]** | Low risk | Low risk | Low risk | Registered | Unclear | High risk | Low risk | Low risk | Low risk | High risk | Unclear risk | High risk |
| **Robinson et al., 2012[43]** | Low risk | Low risk | Low risk | Registered | Unclear | High risk | High risk | Low risk | Unclear risk | High risk | Unclear risk | High risk |
| **Rossouw and Fonagy 2012[14]** | Low risk | Low risk | Low risk | Registered | Published | Low risk | High risk | Low risk | Low risk | High risk | Low risk | High risk |
| **Santamarina-Perez et al., 2020[44]** | Low risk | Low risk | Low risk | Registered | Published | Low risk | Low risk | Low risk | Low risk | High risk | Unclear risk | High risk |
| **Schuppert et al., 2009[45]** | Low risk | Unclear risk | Low risk | Registered | Unclear | High risk | High risk | Low risk | Unclear risk | High risk | High risk | High risk |
| **Schuppert et al., 2012[46]** | Low risk | Low risk | Low risk | Registered | Published | High risk | Unclear risk | Low risk | Low risk | High risk | High risk | High risk |
| **Sinyor et al., 2020[47]** | Low risk | Low risk | Low risk | Unclear | Published | High risk | High risk | Low risk | Low risk | Low risk | High risk | High risk |
| **Tang et al., 2009[12]** | Low risk | High risk | Low risk | Unclear | Unclear | High risk | Low risk | High risk | Unclear risk | Low risk | Unclear risk | High risk |
| **Van Voorhees et al., 2009[48]** | Low risk | Low risk | Low risk | Registered | Unclear | High risk | Low risk | Low risk | Low risk | High risk | Unclear risk | High risk |
| **Wharff et al. 2019[49]** | Low risk | Low risk | Low risk | Unclear | Unclear | High risk | High risk | Low risk | Low risk | High risk | Unclear risk | High risk |
| **Wood et al. 2001[50]** | Low risk | Low risk | Low risk | Not registered | Unclear | Unclear risk | Low risk | Low risk | Low risk | High risk | Low risk | High risk |
| **Yen et al. 2019[51]** | Low risk | Low risk | Low risk | Not registered | Unclear | Unclear risk | Low risk | Low risk | Unclear risk | High risk | Unclear risk | High risk |

© 2021 Bahji A et al. *JAMA Network Open*.

**eReferences**


1. Goodyer IM, Reynolds S, Barrett B, et al. Cognitive behavioural therapy and short-term psychoanalytical psychotherapy versus a brief psychosocial intervention in adolescents with unipolar major depressive disorder (IMPACT): a multicentre, pragmatic, observer-blind, randomised controlled superiority trial. *Lancet Psychiatry*. 2017;4(2):109-119. doi:10.1016/S2215-0366(16)30378-9

2. Milner A, Spittal MJ, Kapur N, Witt K, Pirkis J, Carter G. Mechanisms of brief contact interventions in clinical populations: a systematic review. *BMC Psychiatry*. 2016;16(1):194. doi:10.1186/s12888-016-0896-4

3. Esposito-Smythers C, Hadley W, Curby TW, Brown LK. Randomized Pilot Trial of a Cognitive-Behavioral Alcohol, Self-Harm, and HIV Prevention Program For Teens in Mental Health Treatment. *Behav Res Ther*. 2017;89:49-56. doi:10.1016/j.brat.2016.11.005

4. Hetrick SE, Yuen HP, Bailey E, et al. Internet-based cognitive behavioural therapy for young people with suicide-related behaviour (Reframe-IT): a randomised controlled trial. *Evid Based Ment Health*. 2017;20(3):76-82. doi:10.1136/eb-2017-102719

5. McCauley E, Berk MS, Asarnow JR, et al. Efficacy of Dialectical Behavior Therapy for Adolescents at High Risk for Suicide: A Randomized Clinical Trial. *JAMA Psychiatry*. 2018;75(8):777-785. doi:10.1001/jamapsychiatry.2018.1109

6. Mehlum L, Tørmoen AJ, Ramberg M, et al. Dialectical Behavior Therapy for Adolescents With Repeated Suicidal and Self-harming Behavior: A Randomized Trial. *J Am Acad Child Adolesc Psychiatry*. 2014;53(10):1082-1091. doi:10.1016/j.jaac.2014.07.003

7. Britton WB, Lepp NE, Niles HF, Rocha T, Fisher N, Gold J. A Randomized Controlled Pilot Trial of Classroom-Based Mindfulness Meditation Compared to an Active Control Condition in 6th Grade Children. *J Sch Psychol*. 2014;52(3):263-278. doi:10.1016/j.jsp.2014.03.002

8. Eclectic Therapy | Psychology Today Canada. Accessed September 20, 2020. https://www.psychologytoday.com/ca/therapy-types/eclectic-therapy

9. Cottrell DJ, Wright-Hughes A, Collinson M, et al. Effectiveness of systemic family therapy versus treatment as usual for young people after self-harm: a pragmatic, phase 3, multicentre, randomised controlled trial. *Lancet Psychiatry*. 2018;5(3):203-216. doi:10.1016/S2215-0366(18)30058-0

10. Diamond GS, Wintersteen MB, Brown GK, et al. Attachment-based family therapy for adolescents with suicidal ideation: a randomized controlled trial. *J Am Acad Child Adolesc Psychiatry*. 2010;49(2):122-131. doi:10.1097/00004583-201002000-00006

11. Harrington R, Kerfoot M, Dyer E, et al. Randomized Trial of a Home-Based Family Intervention for Children Who Have Deliberately Poisoned Themselves. *J Am Acad Child Adolesc Psychiatry*. 1998;37(5):512-518. doi:10.1016/S0890-8567(14)60001-0


© 2021 Bahji A et al. *JAMA Network Open*.

12. Tang T-C, Jou S-H, Ko C-H, Huang S-Y, Yen C-F. Randomized study of school-based intensive interpersonal psychotherapy for depressed adolescents with suicidal risk and parasuicide behaviors. *Psychiatry Clin Neurosci*. 2009;63(4):463-470. doi:10.1111/j.1440-1819.2009.01991.x

13. Griffiths H, Duffy F, Duffy L, et al. Efficacy of Mentalization-based group therapy for adolescents: the results of a pilot randomised controlled trial. *BMC Psychiatry*. 2019;19(1):167. doi:10.1186/s12888-019-2158-8

14. Rossouw TI, Fonagy P. Mentalization-Based Treatment for Self-Harm in Adolescents: A Randomized Controlled Trial. *J Am Acad Child Adolesc Psychiatry*. 2012;51(12):1304-1313.e3. doi:10.1016/j.jaac.2012.09.018

15. Ougrin D, Corrigall R, Poole J, et al. Comparison of effectiveness and cost-effectiveness of an intensive community supported discharge service versus treatment as usual for adolescents with psychiatric emergencies: a randomised controlled trial. *Lancet Psychiatry*. 2018;5(6):477-485. doi:10.1016/S2215-0366(18)30129-9

16. Alavi A, Sharifi B, Ghanizadeh A, Dehbozorgi G. Effectiveness of Cognitive-Behavioral Therapy in Decreasing Suicidal Ideation and Hopelessness of the Adolescents with Previous Suicidal Attempts. *Iran J Pediatr*. 2013;23(4):467-472.

17. Apsche JA, Bass CK, Houston M-A. A one year study of adolescent males with aggression and problems of conduct and personality: A comparison of MDT and DBT. *Int J Behav Consult Ther*. 2006;2(4):544-552. doi:10.1037/h0101006

18. Asarnow JR, Baraff LJ, Berk M, et al. An emergency department intervention for linking pediatric suicidal patients to follow-up mental health treatment. *Psychiatr Serv Wash DC*. 2011;62(11):1303-1309. doi:10.1176/ps.62.11.pss6211_1303

19. Asarnow JR, Hughes JL, Babeva KN, Sugar CA. Cognitive-Behavioral Family Treatment for Suicide Attempt Prevention: A Randomized Controlled Trial. *J Am Acad Child Adolesc Psychiatry*. 2017;56(6):506-514. doi:10.1016/j.jaac.2017.03.015

20. Beck E, Bo S, Jørgensen MS, et al. Mentalization-based treatment in groups for adolescents with borderline personality disorder: a randomized controlled trial. *J Child Psychol Psychiatry*. 2020;61(5):594-604. doi:10.1111/jcpp.13152

21. Jørgensen MS, Storebø OJ, Bo S, et al. Mentalization-based treatment in groups for adolescents with Borderline Personality Disorder: 3- and 12-month follow-up of a randomized controlled trial. *Eur Child Adolesc Psychiatry*. Published online May 9, 2020. doi:10.1007/s00787-020-01551-2

22. Chanen AM, Jackson HJ, McCutcheon LK, et al. Early intervention for adolescents with borderline personality disorder using cognitive analytic therapy: randomised controlled trial. *Br J Psychiatry*. 2008;193(6):477-484. doi:10.1192/bjp.bp.107.048934

© 2021 Bahji A et al. *JAMA Network Open*.

23. Cooney E, New Zealand, Ministry of Health, Wise Group (N.Z.), Te Pou o te Whakaaro Nui. *Feasibility of Evaluating DBT for Self-Harming Adolescents: A Small Randomised Controlled Trial*. Te Pou o Te Whakaaro Nui =The National Centre of Mental Health Research and Workforce Development; 2010. Accessed September 12, 2020. http://www.tepou.co.nz/file/Research-projects/suicide/feasibility-of-evaluating-dbt-for-self-harming-adolescents-report.pdf

24. Cotgrove A, Zirinsky L, Black D, Weston D. Secondary prevention of attempted suicide in adolescence. *J Adolesc*. 1995;18(5):569-577. doi:10.1006/jado.1995.1039

25. Cottrell DJ, Wright-Hughes A, Eisler I, et al. Longer-term effectiveness of systemic family therapy compared with treatment as usual for young people after self-harm: An extended follow up of pragmatic randomised controlled trial. *EClinicalMedicine*. 2020;18:100246. doi:10.1016/j.eclinm.2019.100246

26. Diamond GS, Wintersteen MB, Brown GK, et al. Attachment-Based Family Therapy for Adolescents with Suicidal Ideation: A Randomized Controlled Trial. *J Am Acad Child Adolesc Psychiatry*. 2010;49(2):122-131. doi:10.1016/j.jaac.2009.11.002

27. Diamond GS, Kobak RR, Krauthamer Ewing ES, et al. A Randomized Controlled Trial: Attachment-Based Family and Nondirective Supportive Treatments for Youth Who Are Suicidal. *J Am Acad Child Adolesc Psychiatry*. 2019;58(7):721-731. doi:10.1016/j.jaac.2018.10.006

28. Donaldson D, Spirito A, Esposito-Smythers C. Treatment for Adolescents Following a Suicide Attempt: Results of a Pilot Trial. *J Am Acad Child Adolesc Psychiatry*. 2005;44(2):113-120. doi:10.1097/00004583-200502000-00003

29. Esposito-Smythers C, Spirito A, Kahler CW, Hunt J, Monti P. Treatment of Co-Occurring Substance Abuse and Suicidality Among Adolescents: A Randomized Trial. *J Consult Clin Psychol*. 2011;79(6):728-739. doi:10.1037/a0026074

30. Gleeson JFM, Chanen A, Cotton SM, Pearce T, Newman B, McCutcheon L. Treating co-occurring first-episode psychosis and borderline personality: a pilot randomized controlled trial. *Early Interv Psychiatry*. 2012;6(1):21-29. doi:10.1111/j.1751-7893.2011.00306.x

31. Green JM, Wood AJ, Kerfoot MJ, et al. Group therapy for adolescents with repeated self harm: randomised controlled trial with economic evaluation. *BMJ*. 2011;342:d682. doi:10.1136/bmj.d682

32. Hazell PL, Martin G, Mcgill K, et al. Group therapy for repeated deliberate self-harm in adolescents: failure of replication of a randomized trial. *J Am Acad Child Adolesc Psychiatry*. 2009;48(6):662-670. doi:10.1097/CHI.0b013e3181aOacec

33. Hill RM, Pettit JW. Pilot Randomized Controlled Trial of LEAP: A Selective Preventive Intervention to Reduce Adolescents’ Perceived Burdensomeness. *J Clin Child Adolesc Psychol*. 2019;48(sup1):S45-S56. doi:10.1080/15374416.2016.1188705

© 2021 Bahji A et al. *JAMA Network Open*.

34. Kaess M, Edinger A, Fischer-Waldschmidt G, Parzer P, Brunner R, Resch F. Effectiveness of a brief psychotherapeutic intervention compared with treatment as usual for adolescent nonsuicidal self-injury: a single-centre, randomised controlled trial. *Eur Child Adolesc Psychiatry*. 2020;29(6):881-891. doi:10.1007/s00787-019-01399-1

35. Kennard BD, Goldstein T, Foxwell AA, et al. As Safe as Possible (ASAP): A Brief App-Supported Inpatient Intervention to Prevent Postdischarge Suicidal Behavior in Hospitalized, Suicidal Adolescents. *Am J Psychiatry*. 2018;175(9):864-872. doi:10.1176/appi.ajp.2018.17101151

36. King CA, Kramer A, Preuss L, Kerr DCR, Weisse L, Venkataraman S. Youth-Nominated Support Team for suicidal adolescents (Version 1): A randomized controlled trial. *J Consult Clin Psychol*. 2006;74(1):199-206. doi:10.1037/0022-006X.74.1.199

37. King CA, Klaus N, Kramer A, Venkataraman S, Quinlan P, Gillespie B. The Youth-Nominated Support Team for Suicidal Adolescents – Version II: A Randomized Controlled Intervention Trial. *J Consult Clin Psychol*. 2009;77(5):880-893. doi:10.1037/a0016552

38. King CA, Gipson PY, Horwitz AG, Opperman KJ. Teen Options for Change: An Intervention for Young Emergency Patients Who Screen Positive for Suicide Risk. *Psychiatr Serv*. 2014;66(1):97-100. doi:10.1176/appi.ps.201300347

39. Mehlum L, Ramberg M, Tørmoen AJ, et al. Dialectical Behavior Therapy Compared With Enhanced Usual Care for Adolescents With Repeated Suicidal and Self-Harming Behavior: Outcomes Over a One-Year Follow-Up. *J Am Acad Child Adolesc Psychiatry*. 2016;55(4):295-300. doi:10.1016/j.jaac.2016.01.005

40. Ougrin D, Zundel T, Ng A, Banarsee R, Bottle A, Taylor E. Trial of Therapeutic Assessment in London: randomised controlled trial of Therapeutic Assessment versus standard psychosocial assessment in adolescents presenting with self-harm. *Arch Dis Child*. 2011;96(2):148-153. doi:10.1136/adc.2010.188755

41. Ougrin D, Boege I, Stahl D, Banarsee R, Taylor E. Randomised controlled trial of therapeutic assessment versus usual assessment in adolescents with self-harm: 2-year follow-up. *Arch Dis Child*. 2013;98(10):772-776. doi:10.1136/archdischild-2012-303200

42. Pineda J, Dadds MR. Family Intervention for Adolescents With Suicidal Behavior: A Randomized Controlled Trial and Mediation Analysis. *J Am Acad Child Adolesc Psychiatry*. 2013;52(8):851-862. doi:10.1016/j.jaac.2013.05.015

43. Robinson J, Yuen HP, Gook S, et al. Can receipt of a regular postcard reduce suicide-related behaviour in young help seekers? A randomized controlled trial. *Early Interv Psychiatry*. 2012;6(2):145-152. doi:10.1111/j.1751-7893.2011.00334.x

44. Santamarina-Perez P, Mendez I, Singh MK, et al. Adapted Dialectical Behavior Therapy for Adolescents with a High Risk of Suicide in a Community Clinic: A Pragmatic Randomized Controlled Trial. *Suicide Life Threat Behav*. 2020;50(3):652-667. doi:10.1111/sltb.12612

© 2021 Bahji A et al. *JAMA Network Open*.

45. Schuppert HM, Giesen-Bloo J, van Gemert TG, et al. Effectiveness of an emotion regulation group training for adolescents--a randomized controlled pilot study. *Clin Psychol Psychother*. 2009;16(6):467-478. doi:10.1002/cpp.637

46. Schuppert H, Timmerman ME, Bloo J, et al. Emotion Regulation Training for Adolescents With Borderline Personality Disorder Traits: A Randomized Controlled Trial. *J Am Acad Child Adolesc Psychiatry*. 2012;51(12):1314-1323.e2. doi:10.1016/j.jaac.2012.09.002

47. Sinyor M, Williams M, Mitchell R, et al. Cognitive behavioral therapy for suicide prevention in youth admitted to hospital following an episode of self-harm: A pilot randomized controlled trial. *J Affect Disord*. 2020;266:686-694. doi:10.1016/j.jad.2020.01.178

48. Van Voorhees BW, Fogel J, Reinecke MA, et al. Randomized Clinical Trial of an Internet-Based Depression Prevention Program for Adolescents (Project CATCH-IT) in Primary Care: Twelve-Week Outcomes. *J Dev Behav Pediatr JDBP*. 2009;30(1):23-37. doi:10.1097/DBP.0b013e3181966c2a

49. Wharff E, Ginnis K, Ross A, White E, White M, Forbes P. Family-Based Crisis Intervention With Suicidal Adolescents: A Randomized Clinical Trial. *Pediatr Emerg Care*. 2019;35(3):170-175. doi:10.1097/PEC.0000000000001076

50. Wood A, Trainor G, Rothwell J, Moore A, Harrington R. Randomized trial of group therapy for repeated deliberate self-harm in adolescents. *J Am Acad Child Adolesc Psychiatry*. 2001;40(11):1246-1253. doi:10.1097/00004583-200111000-00003

51. Yen S, Spirito A, Weinstock LM, Tezanos K, Kolobaric A, Miller I. Coping long term with active suicide in adolescents: Results from a pilot randomized controlled trial. *Clin Child Psychol Psychiatry*. 2019;24(4):847-859. doi:10.1177/1359104519843956

© 2021 Bahji A et al. *JAMA Network Open*.
